# C5a Enhances Dysregulated Inflammatory and Angiogenic Responses to Malaria *In Vitro*: Potential Implications for Placental Malaria

**DOI:** 10.1371/journal.pone.0004953

**Published:** 2009-03-24

**Authors:** Andrea Conroy, Lena Serghides, Constance Finney, Simon O. Owino, Sanjeev Kumar, D. Channe Gowda, W. Conrad Liles, Julie M. Moore, Kevin C. Kain

**Affiliations:** 1 McLaughlin-Rotman Centre for Global Health, Toronto General Hospital, McLaughlin Centre for Molecular Medicine, University of Toronto, Toronto, Ontario, Canada; 2 Department of Laboratory Medicine and Pathobiology, University of Toronto, Toronto, Ontario, Canada; 3 Center for Tropical and Emerging Global Diseases and Department of Infectious Diseases, College of Veterinary Medicine, University of Georgia, Athens, Georgia, United States of America; 4 Centre for Global Health Research, Kenya Medical Research Institute, Kisumu, Kenya; 5 Department of Biochemistry and Molecular Biology, Pennsylvania State University College of Medicine, Hershey, Pennsylvania, United States of America; 6 Tropical Disease Unit, Division of Infectious Diseases, Department of Medicine, University Health Network-Toronto General Hospital, Toronto, Ontario, Canada; Instituto Oswaldo Cruz and FIOCRUZ, Brazil

## Abstract

**Background:**

Placental malaria (PM) is a leading cause of maternal and infant mortality. Although the accumulation of parasitized erythrocytes (PEs) and monocytes within the placenta is thought to contribute to the pathophysiology of PM, the molecular mechanisms underlying PM remain unclear. Based on the hypothesis that excessive complement activation may contribute to PM, in particular generation of the potent inflammatory peptide C5a, we investigated the role of C5a in the pathogenesis of PM *in vitro* and *in vivo*.

**Methodology and Principal Findings:**

Using primary human monocytes, the interaction between C5a and malaria *in vitro* was assessed. CSA- and CD36-binding PEs induced activation of C5 in the presence of human serum. *Plasmodium falciparum* GPI (*pf*GPI) enhanced C5a receptor expression (CD88) on monocytes, and the co-incubation of monocytes with C5a and *pf*GPI resulted in the synergistic induction of cytokines (IL-6, TNF, IL-1β, and IL-10), chemokines (IL-8, MCP-1, MIP1α, MIP1β) and the anti-angiogenic factor sFlt-1 in a time and dose-dependent manner. This dysregulated response was abrogated by C5a receptor blockade. To assess the potential role of C5a in PM, C5a plasma levels were measured in malaria-exposed primigravid women in western Kenya. Compared to pregnant women without malaria, C5a levels were significantly elevated in women with PM.

**Conclusions and Significance:**

These results suggest that C5a may contribute to the pathogenesis of PM by inducing dysregulated inflammatory and angiogenic responses that impair placental function.

## Introduction

Placental malaria (PM) is a major determinant of maternal and infant health in the developing world. PM, especially in primigravidae, can have profound maternal and fetal health consequences, including anemia, stillbirth, premature delivery, intrauterine growth restriction (IUGR) and delivery of low birth weight (LBW) infants [1], [2]. The accumulation of *Plasmodium falciparum* parasitized erythrocytes (PEs) within the placenta is believed to be an essential step in the pathogenesis of PM [3]. A subpopulation of PEs that express novel variant surface antigens (VSA-PM), specifically adhere to glycosaminoglycan chondroitin sulfate A (CSA) in the placental intervillous space [3]–[5].

PM is also characterized by the infiltration of the placenta with maternal mononuclear cells (mφ). The sequestered PEs release bioactive molecules including those with associated glycosylphosphatidylinositol anchor molecules (*pf*GPI) that can stimulate maternal mφ [6]–[10] and fetal syncytiotrophoblast [6], [11], [12] to produce inflammatory cytokines, such as TNF and IFN-γ, and β-chemokines including macrophage-inflammatory protein (MIP)-1α, MIP-1β, monocyte chemoattractant protein-1 (MCP-1), and macrophage migration inhibitory factor (MIF). These cytokines and chemokines further recruit, retain, and activate mφ in the placenta [6]–[8], [12], [13]. The resultant accumulation of activated mφ is believed to contribute to adverse birth outcomes [14]–[16]. Although PEs and mφ collect in the placenta, how they may contribute to fetal and placental injury is unknown.

The maintenance of regulated cytokine responses in the placenta is essential in order to prevent rejection of the semiallogenic fetoplacental unit and ensure appropriate immunological responses to infection [17]. This involves a generalized physiological adaptation that results in a bias towards cytokines promoting humoral immunity at the expense of cell-mediated immunity [18]. PM is associated with heightened T_H_^1^-type responses that disrupt the balance of cytokines at the maternal-fetal interface [14], [16], [19], [20]. However, the molecular basis underlying PM-associated cytokine dysregulation, placental immunopathology, and adverse birth outcomes is incompletely understood.

The complement system is an essential component of the innate immune response to a number of infectious agents. The complement cascade can be activated by four distinct pathways, three of which converge at the level of the C3 component, leading to the cleavage of C3 and C5 to their activated forms, C3a and C5a, as well as the formation of the terminal membrane attack complex [21], [22]. Recently, a pathway of C5a generation occurring independently of C3 was described [21], [23]. Several lines of evidence have implicated excessive activation of the complement system, notably generation of the potent pro-inflammatory peptide C5a, in mediating deleterious innate host responses to bacterial and fungal infections and contributing to the development of sepsis (reviewed in [22]) [21]–[26]. Sepsis, similar to severe malaria, is a clinical syndrome characterized by systemic inflammation and endothelial activation in response to infection [27], [28]. Elevated C5a levels in sepsis have been implicated in adverse clinical outcomes and death. Blockade of C5a-C5aR activity in animal models of sepsis prevents end-organ injury and improves survival [29]–[31].

Excessive C5a generation has also recently been identified as a critical mediator of placental and fetal injury in a non-infectious mouse model of spontaneous miscarriage and IUGR, via dysregulation of angiogenic factors required for normal placental development [32]. Activated complement components were shown to directly induce the release of soluble fms-like tyrosine kinase-1 (sFlt-1, sVEGFR-1), a potent anti-angiogenic factor that prevents signaling of vascular endothelial growth factor and placental growth factor [33]. Excessive levels of sFlt-1 inhibit placental differentiation and are thought to play a direct role in pre-eclampsia [34]–[36] and C5a-associated IUGR and pregnancy loss [32]. Of note, Muehlenbachs et al. have reported elevated plasma levels of sFlt-1 in primigravid women with PM [37].

Based on the hypothesis that C5a may play a role in PM by generating angiogenic factors that mediate IUGR and fetal loss, we determined whether malaria parasites can activate C5 and if C5a potentiates the induction of sFlt-1, inflammatory cytokines and chemokines associated with adverse pregnancy outcomes. Here we show that CSA- and CD36-binding PEs activate C5, that C5a and *pf*GPI cooperate to induce an amplified inflammatory and anti-angiogenic response to *P. falciparum* malaria *in vitro*, and that primigravid women with PM have elevated circulating C5a levels *in vivo*.

## Methods

### *P. falciparum* culture

The laboratory isolates CS2 (CSA-binding) and E8B (ICAM-1 and CD36 binding) were cultured in vitro as described [38], [39]. Cultures were routinely treated with mycoplasma removal agent (ICN), and were tested and found negative for mycoplasma by PCR.

### Mononuclear cell isolation

PBMCs were isolated from healthy volunteers using a Ficoll gradient as previously described [40]. PBMCs were counted using a haemocytometer and cell viability was determined by Trypan blue exclusion test. Cells were plated at a concentration of 2.0×10^6^ cells/mL (48 well plate) in RPMI 1640 medium supplemented with 10% heat inactivated FBS and gentamycin (R10G). Cells were cultured at 37°C in 5% CO_2_ for the time indicated.

### Isolation and purification of GPIs from *P. falciparum*

HPLC-purified *P. falciparum* GPI (*pf*GPI) was isolated [41], conjugated to gold beads and used at a concentration of 300 ng/ml as previously described [10]. Unconjugated gold beads alone were added as controls to wells containing media, or C5a (endotoxin-free; Biovision, USA). All preparations of purified *pf*GPIs were tested for endotoxin by Limulus amebocyte lysate assay prior to use.

### Complement assays

PEs, uninfected red blood cells (uRBCs), or media control were incubated in the presence of 30% serum from malaria naïve donors. After 30 min, supernatants were collected and analyzed for C5 activation. C5a levels in culture supernatants and human plasma were measured by ELISA (R&D Systems).

### Cytokine and chemokine assays

PBMCs were cultured with or without recombinant human C5a in addition to *P. falciparum* culture supernatants (1∶10 dilution) and R10G alone as media control, or HPLC-purified GPI (300 ng/mL) and unconjugated gold beads as a control. C5a concentrations ranging from 1 nM to 100 nM were selected representing a spectrum of C5a levels ranging from physiologically normal levels (<10 nM) to those associated with sepsis (10–100 nM) [25], [42]. For receptor blockade studies *pf*GPI, the C5aR blocking antibody (Serotec, S5/1, 5 µg/mL) or an isotype control (eBioscience, eBM2a, 5 µg/mL) was added to culture medium just prior to stimulation with 50 nM C5a. Culture supernatants were collected aseptically at times indicated. IL-6, TNF, IL-1β, and IL-10 were measured by Cytometric Bead Array (CBA) (BD Biosciences, human inflammation kit). IL-8, monocyte chemoattractant protein-1 (MCP-1), and macrophage inflammatory protein-1α and - β (MIP-1α and MIP-1β) and sFlt-1 (sVEGFR-1) were measured by ELISA (R&D Systems).

### Flow cytometry

PBMCs (2.0×10^6^) were blocked in 20% serum for 20 min at 4°C. Cells were then stained for CD14-APC (eBioscience, 61D3, 100 uL/mL) and CD88-PE or isotype IgG1-PE (BD Biosciences, D53-1473, 150 uL/mL and eBioscience, P3, 150 uL/mL) for 30 min at 4°C. Cells were fixed in 1% paraformaldehyde/PBS overnight and analyzed on a Becton Dickinson FACS Calibur flow cytometer. Data was analyzed using FlowJo software. A minimum of 50,000 events were collected. Positive background PE staining in isotype samples was subtracted from respective CD88-PE stained samples and then reported as specific CD88 staining.

### Statistical analysis

Data are expressed as means±SEM, unless otherwise noted. For continuous variables a Student's t test or one-way ANOVA with Bonferonni post-tests was used to compare means. Non-parametric tests (Mann-Whitney or Kruskall-Wallis) were used to compare groups where parametric test conditions were not met. Synergism was determined by the interaction term of a two way ANOVA (C5a*GPI). Synergism in the time course was determined using the cumulative cytokine levels after 48 hours by performing a two way ANOVA and examining the interaction term. Significance in the C5a receptor blockade experiments was assessed by repeated measures ANOVA to determine whether levels of cytokines differed between cells treated with *pf*GPI alone, or anti-CD88 plus C5a and *pf*GPI, or student's t-test by comparing the isotype and anti-CD88 co-treated (C5a and *pf*GPI) groups after correcting for the background levels observed in media controls. In cases where C5a receptor blockade resulted in values of “0”, a one sample t-test was used to determine whether the isotype C5a and *pf*GPI co-treated groups differed from a hypothetical value of “0”. Correlation of peripheral parasitemia to placental parasitemia was determined using Spearman correlation. Experiments were performed in duplicate or triplicate and repeated as indicated in the figure legends. Statistical analysis of experimental replicates is found in the supporting information files (Supporting Information S1, S2, S3, S4). Differences with p<0.05 were considered significant. Statistical analysis was performed using GraphPad Prism and SPSS.

### Study participants

Plasma samples were collected at time of delivery from the peripheral blood and placental blood of primigravid women, living in an area of holoendemic malaria transmission in Western Kenya, with and without placental malaria infection as described previously [7]. Malaria infection was evaluated by examination of Giemsa-stained peripheral and placental thick and thin blood smears by light microscopy. Percent parasitemia was estimated by counting a minimum of 1000 erythrocytes and calculated using the following formula: (# infected erythrocytes/total # erythrocytes) ×100. Women who tested positive for HIV were excluded. This study was approved by the institutional review boards of the University of Georgia, the Kenya Medical Research Institute and the Centers for Disease Control and Prevention, Atlanta, Georgia, United States. Written informed consent was obtained from all participants.

## Results

### CSA- and CD36-binding PEs activate C5 and *pf*GPI induces C5a receptor expression

**Figure 1:**
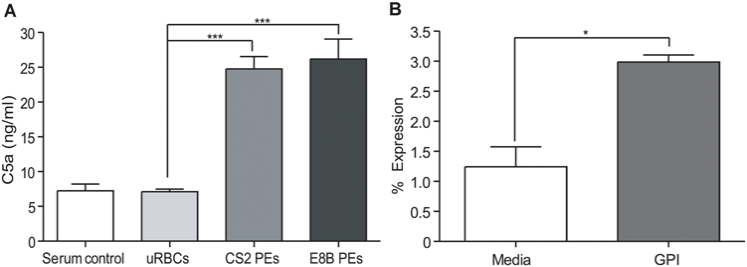
Parasitized erythrocytes (PEs) induce C5 activation and *pf*GPI upregulates C5aR expression on monocytes. (A) Serum from malaria-naïve donors was added to mature stage CS2 (CSA binding) PEs or E8B (ICAM-1 and CD36 binding), uninfected red blood cells (uRBCs) or media for 30 min and supernatants were assayed for C5a by ELISA. Increased C5a levels were observed in the supernatants of PEs compared to uRBC or control, (Student's t-test: uRBCs vs. Mature PEs ***p<0.001). Data are presented as means±SEM and are representative of four independent experiments for CS2 parasites and two independent experiments for CD36-binding parasites. (B) Human PBMCs were stimulated with *pf*GPI or unconjugated gold beads alone as a control for 24 h. Expression of C5a receptor was determined by flow cytometry on monocytes (CD14). The percentage of monocytes expressing C5aR was significantly higher for *pf*GPI-treated versus media control cells, (Student's t-test: *p = 0.0279). Data are presented as means±SEM and are representative of two independent experiments.

The pro-inflammatory anaphylatoxin C5a has been implicated in mediating deleterious host responses to bacterial and fungal agents [22], [25]. To confirm that CSA-binding PEs were capable of activating the complement system and thus generating C5a, serum from malaria-naïve donors was added to mature stage CS2 PEs or uRBCS. Compared to uRBCs or controls, C5 activation was only observed in the presence of PEs (**Figure 1A**) (mean C5a levels: CS2 PEs 24.77 ng/mL [95% confidence interval (CI) 2.229–47.30], E8B PEs 26.19 ng/ml [95% CI −10.44–62.82], uRBCs 7.114 ng/mL [95% CI 5.922–8.306], control 7.242 ng/mL [95% CI 4.116–10.37]; CS2 PEs vs. uRBCs p<0.001 Student's t-test). Non-PM PEs (E8B) also activated C5 (E8B PEs vs. uRBCs p<0.001 Student's t-test).

In order to respond to activated C5, cells must express a C5a receptor. Previous studies report that C5a responsiveness increases following cellular stimulation [43]. To determine the effect of *pf*GPI on monocyte C5aR (CD88) expression, PBMCs were incubated as above with *pf*GPI or media control for 24 or 48 hours, and C5aR and CD14 expression (used to define monocytes) was determined by flow cytometry. Compared to media only control cells, a significant increase in the number of monocytes expressing C5aR (**Figure 1B**) was observed (mean: media 1.2% [95% CI −0.2–2.7] vs. *pf*GPI 3.0 [95% CI 1.5–4.4] p = 0.0279 by Student's t-test). The density of the C5aR, as determined by the mean fluorescent intensity on monocytes already expressing the receptor, was unchanged (data not shown).

### C5a potentiates *pf*GPI-induced inflammatory responses

**Figure 2:**
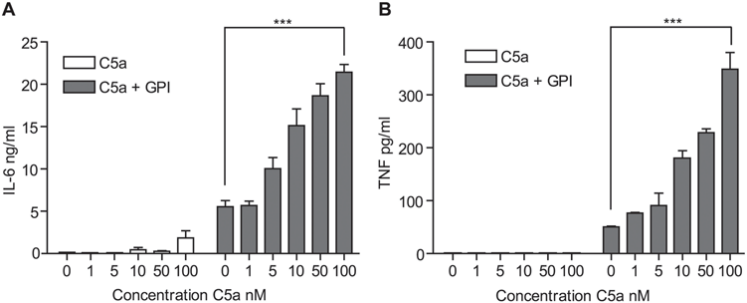
C5a potentiates *pf*GPI-induced inflammatory cytokines in a dose-dependent manner. Human PBMCs were stimulated with varying concentrations of recombinant human C5a (0, 1, 5, 10, 50, 100 nM) for 24 hours, with or without *pf*GPI. Culture supernatants were collected and assayed for IL-6 (A) and TNF (B) by ELISA. Data were analyzed by two-way ANOVA and demonstrate an interaction effect (C5a*pfGPI) for IL-6 (***p<0.0001) and for TNF (***p<0.0001). Data are presented as means±SEM of three independent experiments.

C5a has been shown to enhance TLR-mediated pro-inflammatory responses to microbial products such as LPS, contributing to sepsis syndromes [22], [25], [44], [45]. The parasite product *pf*GPI is recognized by TLR2 and is believed to be a mediator of the inflammatory response that characterizes severe malarial syndromes [10], [44]–[46]. To assess whether C5a would amplify *pf*GPI-induced pro-inflammatory responses, we treated PBMCs with increasing concentrations of recombinant human C5a (0, 1, 5, 10, 50, and 100 nM) with or without *pf*GPI for 24 hours and determined the production of IL-6 and TNF by ELISA. We observed a dose-dependent potentiation of *pf*GPI-induced IL-6 and TNF production by C5a (**Figure 2A and B**; two-way ANOVA (C5a*GPI), p<0.0001).

### C5a Receptor blockade inhibits enhanced cytokine responses

**Figure 3:**
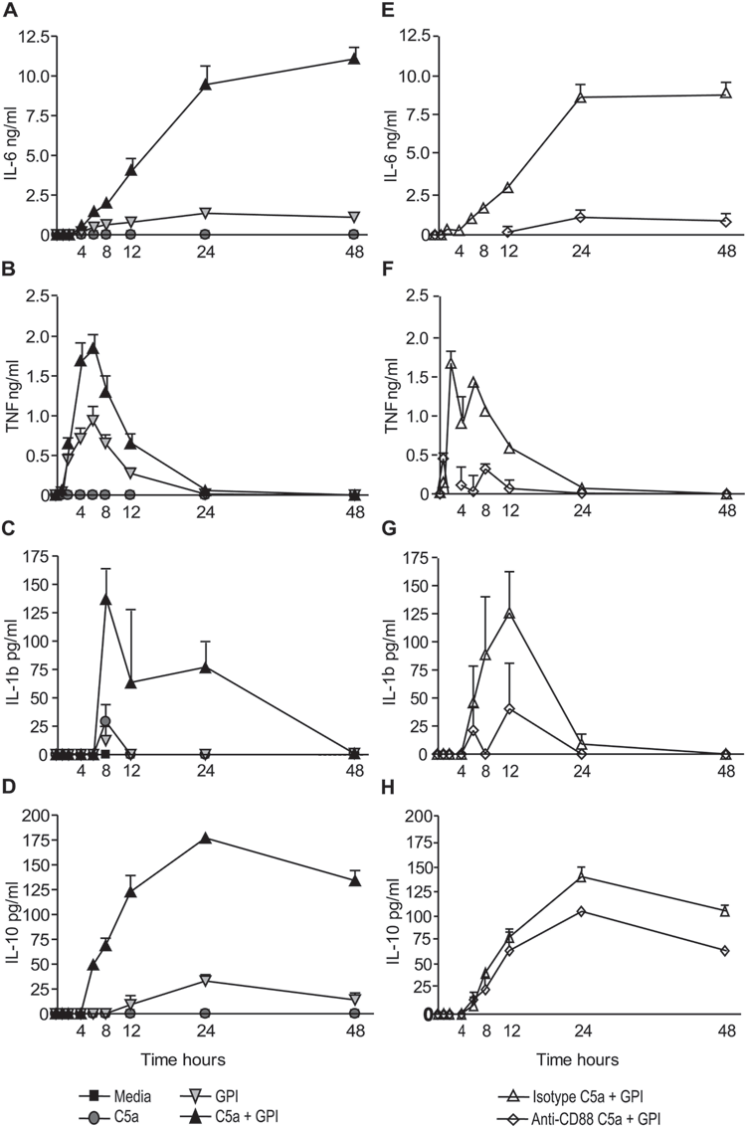
C5a potentiates *pf*GPI-induced inflammatory cytokines and this effect is abrogated by C5a receptor blockade. Human PBMCs were cultured with unconjugated gold beads (media control), unconjugated gold beads and C5a (50 nM), *pf*GPI (300 ng/mL), or a combination of C5a and *pf*GPI. (A–D) Supernatants were assayed for IL-6 (A), TNF (B), IL-1β (C), and IL-10 (D). A significant interaction effect between C5a and *pf*GPI was observed for all cytokines tested. p<0.0001 for IL-6, p = 0.0124 for TNF, p = 0.0418 for IL-1β and p<0.0001 for IL-10 (by two-way ANOVA on cumulative cytokine production over 48 h). Data are representative of three independent experiments. (E–H) Human PBMCs were treated with an α-CD88 mAb (5 µg/mL) to block the C5a receptor or an appropriate isotype control and exposed to either unconjugated gold beads (media control) or a combination of C5a (50 nM) and *pf*GPI (300 ng/mL) for 48 h. Supernatants were assayed for IL-6 (E), TNF (F), IL-1β (G) and IL-10 (H). Cytokine production was significantly reduced upon C5a receptor blockade; p = 0.0005 for IL-6, p = 0.0028 for TNF, p = 0.0479 for IL-1β and p = 0.0191 for IL-10 (repeated measures ANOVA comparing cytokine levels between isotype control mAb and α-CD88 mAb groups co-treated with C5a and *pf*GPI). Values shown are corrected for background levels observed in the media controls, and are presented as means±SEM. Data are representative of two independent experiments.

Elevated levels of T_H_^1^ cytokines have been implicated in PM pathogenesis and adverse pregnancy outcomes [14], [16], [47]. In order to determine the effect of C5a on the kinetics of *pf*GPI-induced cytokine production, we treated PBMCs with C5a (50 nM) and *pf*GPI (HPLC-purified, 300 ng/mL) for 48 hours. Supernatants were collected at baseline, 1, 2, 4, 6, 8, 12, 24 and 48 hours and assayed for inflammatory cytokines using a cytometric bead array. To examine the role of C5a-C5aR interactions in the observed inflammatory responses, we also performed parallel experiments including monoclonal antibody blockade of the C5a receptor. *pf*GPI-stimulated cytokines, including IL-6, TNF, IL-1β and IL-10, were synergistically induced in the presence of C5a (**Figure 3A–D** **:** two-way ANOVA on cumulative cytokine levels (C5a*GPI); p<0.0001 for IL-6, p = 0.0124 for TNF, p = 0.0418 for IL-1β and p<0.0001 for IL-10). A significant reduction in cytokine production was observed in experiments using anti-C5aR blocking antibody compared to its isotype control for all cytokines measured (**Figure 3E–H** **:** repeated measures ANOVA). Further analysis (repeated measures ANOVA) was performed to determine whether C5aR blockade returned cytokine production to levels for cells treated with *pf*GPI alone. In the presence of anti-C5aR, production of IL-6, TNF, and IL-1β was returned to levels that were not significantly different from *pf*GPI alone (p>0.05). However, this was not true for IL-10 (p = 0.0101).

### C5a potentiates secretion of chemokines and the angiogenesis inhibitor sFlt-1

**Figure 4:**
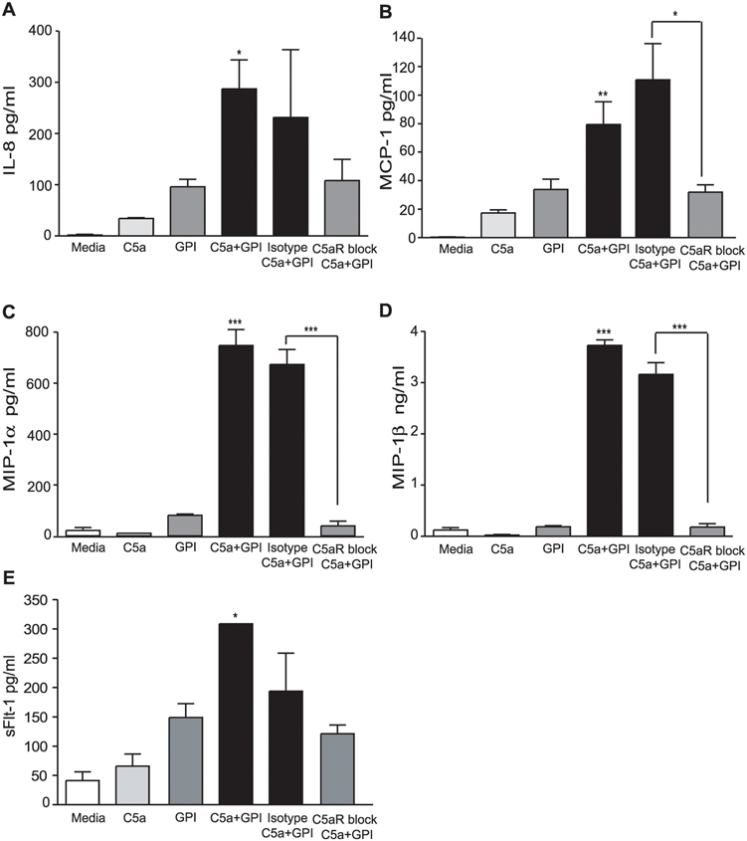
C5a potentiates secretion of *pf*GPI-induced chemokines and the anti-angiogenic factor sFlt-1. Human PBMCs were treated with either unconjugated gold beads (media control), unconjugated gold beads and C5a (50 nM), *pf*GPI (300 ng/mL), or a combination of C5a and *pf*GPI for 48 h. Where indicated PBMCs were treated with either 5 µg/mL of α-CD88 mAb to block the C5a receptor or an appropriate isotype control and were exposed to C5a and *pf*GPI. Supernatants were assayed for IL-8 (A), MCP-1 (B), MIP-1α (C), MIP-1β (D) and sFlt-1 (E). A significant interaction effect between C5a and *pf*GPI was observed; *p = 0.0265 for IL-8, **p = 0.0010 for MCP-1, * p = 0.0177 for sFlt-1, and ***p<0.0001 for MIP-1 α, and MIP-1β (by two-way ANOVA). C5a receptor blockade significantly reduced the levels of MCP-1 (* p = 0.0388), MIP-1α (*** p = 0.0005), and MIP-1β (*** p = 0.0002) (by student's t-test). Data are presented as means±SEM and are representative of three independent experiments.

The infiltration of monocytes into the placenta during PM is associated with adverse birth outcomes [15], [48], [49]. Furthermore, elevated levels of the anti-angiogenic factor sFlt-1 (sVEGFR-1) have been causally implicated in placental dysfunction, growth restriction and fetal loss [32], [37]. In order to determine whether C5a could potentiate the induction of chemokines involved in the recruitment of monocytes and the anti-angiogenic factor sFlt-1, we incubated PBMCs with C5a, *pf*GPI, or media controls with or without C5aR blocking antibody or an isotype control antibody and measured IL-8 (CXCL8), MCP-1 (CCL2), MIP-1α (CCL3), MIP-1β (CCL4), and sFlt-1 (sVEGFR-1) by ELISA (**Figure 4**). In the C5a and *pf*GPI co-treated group, synergistic induction of all factors was observed by two-way ANOVA (C5a*GPI; p = 0.0265 for IL-8, p = 0.0010 for MCP-1, and p<0.0001 for MIP-1 α, MIP-1β and sFlt-1). Blocking C5aR inhibited the augmented secretion of all chemokines with the exception of IL-8 (**Figure 4A–E** **:** Student's t-test p = 0.0388for MCP-1, p = 0.0005 for MIP1-α, p = 0.0002 for MIP1-β). Blockade of the C5aR reduced levels of sFlt-1, but the differences did not consistently reach statistical significance (see supporting information file S4).

### C5a is increased in PM and is associated with higher placental parasite burdens

**Figure 5:**
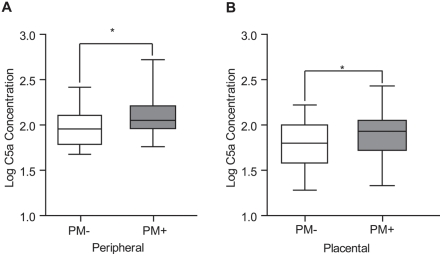
Primigravid women with placental malaria (PM) have increased levels of C5a in peripheral and placental plasma. C5a levels were assayed in peripheral and placental plasma by ELISA. Samples were obtained from HIV negative primigravid women at time of delivery in Kisumu, western Kenya. C5a levels were significantly higher in (A) peripheral plasma of placenta malaria positive (PM+, n = 21) vs. placenta malaria negative (PM−, n = 45) women, *p = 0.01 by two-tailed Student's t-test on log transformed data; and (B) in placenta plasma of placenta malaria positive (PM+, n = 24) vs. placenta malaria negative (PM−, n = 47) women, *p = 0.0264 by Student's t-test two-tailed test on log transformed data.

In order to extend our observations to the *in vivo* setting, we examined C5a levels in malaria-exposed women (**Table 1** **and** **2**). We analyzed C5a levels in the peripheral and placental plasma of HIV negative primigravid women with or without PM from Kisumu, Kenya. Peripheral C5a levels were significantly increased in women with PM (**Figure 5A**: mean C5a levels; PM negative: 98.85 ng/mL [95% CI 84.14–113.6] vs. PM positive: 147.1 ng/mL [95% CI 96.81–197.3] p = 0.01 by Student's t-test two-tailed test on log transformed data). Furthermore, C5a was significantly elevated in the placental plasma of women with PM (**Figure 5B**: mean C5a levels; PM negative: 69.07 ng/mL [95% CI 58.17–79.98] vs. PM positive: 104.0 ng/mL [95% CI 73.10–135.0] p = 0.0264 by Student's t-test two-tailed test on log transformed data). No significant differences were observed between the ages of PM positive vs. PM negative women; however, primigravid women with PM had significantly lower hemoglobin levels, consistent with the destruction of maternal erythrocytes associated with malarial infection (**Table 1** **and** **2**).

**Table 1:** Characteristics of women tested for C5a in peripheral blood.

Group (n)	Age	Hemoglobin	% Parasitemia		Hemozoin (% Score)
	mean±SD	mean±SD	Peripheral	Placental	mean±SD
			mean±SD	mean±SD	
Control (47)	19.04±2.64	11.89±2.92	NA	NA	0±0.00
Malaria (24)	18.48±2.48	9.95±2.37 †	1.54±1.90	5.70±10.25	7.07±8.47

Abbreviations: Hemoglobin (g/dL), % parasitemia (% infected: non-infected erythrocytes), Hemozoin % Score (# pigment containing WBC/ 300WBC).

† p<0.001 (Student's t-test).

**Table 2:** Characteristics of women tested for C5a in placental blood.

Group (n)	Age	Hemoglobin	% Parasitemia		Hemozoin (% Score)
	mean±SD	mean±SD	Peripheral	Placental	mean±SD
			mean±SD	mean±SD	
Control (45)	18.58±2.74	12.36±2.75	NA	NA	0.05±0.34
Malaria (21)	18.13±2.03	10.36±3.09)†	1.41±1.79	4.44±6.46	7.95±11.98

Abbreviations: Hemoglobin (g/dL), % parasitemia (% infected: non-infected erythrocytes), Hemozoin % Score (# pigment containing WBC/ 300WBC).

† p<0.001 (Student's t-test).

## Discussion

This study provides the first evidence implicating excessive activation of the complement component C5 and the induction of anti-angiogenic factors in the pathogenesis of PM. We demonstrate that PEs and parasite products (*pf*GPI) activated C5 and induced C5a receptor expression (**Figure 1**). Further, C5a and *pf*GPI induced synergistic production of inflammatory cytokines, chemokines and an anti-angiogenic factor associated with adverse pregnancy outcomes (**Figures 2** **,** **3** **&** **4**). A role for C5a-C5aR was confirmed by inhibiting inflammation with C5aR blockade (**Figures 3** **&** **4**). These findings were extended to the *in vivo* setting, where primigravid women with PM were shown to have elevated C5a levels in both peripheral and placental plasma (**Figure 5**).

The generation of C5a is normally tightly controlled and, under physiological conditions, C5a enhances the effector function of macrophages and neutrophils, contributing to effective innate responses to microbial pathogens [22]. However, detectable systemic C5a suggests a loss of regulation of complement activation. Elevated C5a levels are associated with a number of deleterious impacts on host innate defence, including defects in phagocyte and endothelial cell function, potentiated chemokine and cytokine secretion to bacterial components such as LPS, and lymphoid apoptosis (reviewed in [22], [25]).

There are several mechanisms by which malaria may induce excessive complement activation, thereby contributing to the pathophysiologic mechanisms underlying PM. We observed that mature stage PEs are capable of generating C5a and a bioactive product of parasite rupture, *pf*GPI can result in an increase in monocytes expressing the C5aR. These observations suggest the potential for an autocrine loop, resulting in augmented inflammatory and angiogenic responses that may act in concert to mediate or amplify placental and fetal injury.

Our observations of malaria-induced C5a are consistent with studies of severe malaria and experimental malaria challenge models that report activation of the complement system [50]–[52]. In experimental human malaria infection, complement activation was observed prior to microscopic detection of parasitemia [50]. Similarly, a recent study of *P. berghei* ANKA implicated C5a as an early mediator of experimental cerebral malaria [53]. In the latter study, C5a levels were elevated prior to the onset of symptoms suggesting a role for C5a as an initiating or amplifying factor in malaria pathogenesis. Our observations are further supported by the findings of a recent genome-wide analysis of PM, where selected complement genes as well as C5aR were shown to be upregulated [54].

During sepsis the excess activation of C5 has been associated with enhanced secretion of pro-inflammatory mediators in response to TLR ligands such as LPS [55]. Similarly *pf*GPI has been shown to induce inflammatory mediators in a TLR2-dependant manner [10], [56]. Additional studies will be needed to determine if the synergistic induction of inflammatory and angiogenic factors observed with *pf*GPI-C5a are also TLR-dependent.

In our *in vitro* model, C5aR blockade abrogated the potentiated inflammatory responses that are believed to be central to the pathogenesis of PM. However, a different response was observed for IL-10. While IL-10 production was reduced by C5aR blockade, the levels did not return to those observed with *pf*GPI alone. We speculate that this may be attributable to the expression of the alternate C5a receptor, C5L2, which has been shown to promote T_H_^2^ skewing [57] as well as have inflammatory effects in experimental sepsis [58]. Future studies will be required to investigate the role of *pf*GPI and other parasite products on the expression of C5L2. When cells were treated with *pf*GPI and C5a, IL-10 was induced earlier than with *pf*GPI alone (**Figure 3D**). This may have important implications *in vivo* since TGF-β and IL-10 regulate inflammatory responses. Early IL-10 may impair T_H_^1^-mediated parasite clearance during malaria infection and increased IL-10 levels have been reported in PM [59].

Monocyte infiltration into the placental intervillous space has been associated with poor birth outcomes including the risk of low birth weight infants [15], [48], [49], [60], [61]. Chemokines function as specific chemoattractants for leukocytes. IL-8 plays a role in neutrophil and monocyte migration, while the β-chemokines are involved in macrophage migration. High levels of macrophage-derived IL-8 during PM have been associated with IUGR [47]. Elevated levels of MIP-1α, MIP-1β and MCP-1 have been observed in PM and it is likely that these chemokines are derived from both maternal (leukocyte) [6], [8] and fetal (syncytiotrophoblast) sources [11], [62]. As with cytokines, C5a and *pf*GPI synergistically induced chemokines, and this effect was abrogated for the β-chemokines by blocking the C5aR (**Figure 4A–D**). No significant difference for IL-8 production was found between cells treated with anti-C5aR versus an isotype control due to the variation in the latter group. However, there was no difference between cells treated with *pf*GPI alone or with anti-C5aR plus C5a and *pf*GPI, indicating that blocking the C5aR inhibits the effect attributable to C5a.

Peak prevalence of malaria infection during pregnancy occurs between 13–20 weeks gestation and gradually falls as gestation increases [63]. This coincides with the second wave of trophoblast invasion of the maternal spiral arteries (16–18 weeks). It is hypothesized that PM may impair transformation of the maternal vasculature leading to placental dysfunction [64], [65]. One protein that may play a role in impaired angiogenesis is the anti-angiogenic factor sFlt-1. This alternatively spliced soluble variant of VEGFR-1 binds and sequesters placental growth factor and vascular endothelial growth factor. Excess sFlt-1 has been shown to inhibit placental cytotrophoblast differentiation and invasion [33]. Increased sFlt-1 levels have been implicated in the pathogenesis of placentation associated with pre-eclampsia and IUGR [34]–[36] and have been shown to predict the development of pre-eclampsia [35]. sFlt-1 can be produced extra-placentally by monocytes treated with C5a [32]. Therefore, we determined whether C5a and *pf*GPI would potentiate sFlt-1 production from mononuclear cells. We show that sFlt-1 was synergistically induced by co-stimulation with these molecules and that this effect was reduced but not consistently inhibited by C5aR blockade (**Figure 4E**). We postulate this may be due to different PBMC donors, as *FLT1* genotype has been associated with differing sFlt-1 levels in the peripheral blood of primigravid women, as well as PBMC responsiveness to LPS *in vitro* [66]. Furthermore, the study by Muehlenbachs et al., provides the first evidence that both fetal and maternal genotype may contribute to immune responses to PM [66]. Taken together, these data suggest a putative mechanism for impaired angiogenesis and placental dysfunction during PM.

In order to extend these observations to human infection, we measured C5a levels in the peripheral blood of a cohort of malaria-exposed primigravid Kenyan women and we measured C5a levels in the placental blood of a similar cohort of malaria-exposed primigravid women at time of delivery (**Table 1** **–** **2**) Significantly increased levels of C5a were found in peripheral blood **(** **Figure 5A** **)** and the placental blood **(** **Figure 5B** **)** of pregnant women with PM compared to those without. These results provide the first evidence that C5a levels are elevated in women with PM. However, these findings will need to be confirmed in larger prospective clinical studies. It will be of interest to also examine how C5a levels change with respect to other disease parameters, including parasitemia and mononuclear cell infiltrates in larger populations.

**Figure 6:**
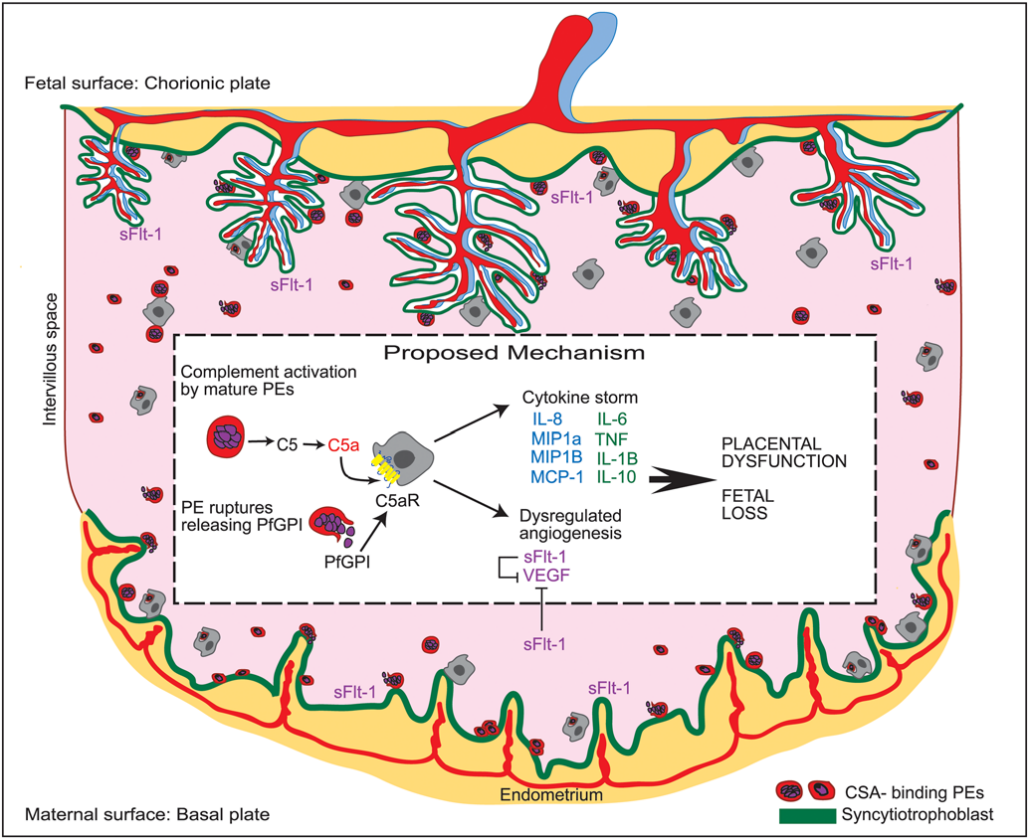
Proposed mechanism for C5a-mediated dysregulation of the placental environment in PM. Mature schizonts activate C5 and rupture releasing parasite components containing GPI that induce expression of C5aR and activate macrophages. The concomitant expression of C5aR and generation of C5a represent a potential autocrine mechanism by which C5a can amplify inflammation in *P. falciparum* malaria resulting in the excessive cytokine and chemokine responses that characterize PM. The dysregulated production of cytokines, chemokines and the anti-angiogenic factor sFlt-1 contribute to placental dysfunction, intrauterine growth restriction and low birth weight infants.

In summary, we have demonstrated a role for C5a in the enhanced cytokine and chemokine responses that characterize PM and in the induction of sFlt-1, an anti-angiogenic factor associated with abnormal placental development and poor birth outcomes (**Figure 6**). Our findings that disruption of the C5a-C5aR interaction inhibits inflammatory and anti-angiogenic responses to malaria suggest anti-C5a-C5aR strategies as potential therapeutic approaches for PM [67].

## Supporting Information

Supporting Information S1 Experimental replicates and statistics Figure 1A and B
(0.03 MB DOC)

Supporting Information S2 Experimental replicates and statistics Figure 2B (TNF)
(0.03 MB DOC)

Supporting Information S3 Experimental replicates and statistics Figure 3
(0.04 MB DOC)

Supporting Information S4 Experimental replicates and statistics Figure 4
(0.05 MB DOC)
