# Supplementary material for: C5a Enhances Dysregulated Inflammatory and Angiogenic Responses to Malaria In Vitro: Potential Implications for Placental Malaria
Source: PLoS One. 2009 Mar 24;4(3):e4953. doi: 10.1371/journal.pone.0004953 (PMC2655724; doi:10.1371/journal.pone.0004953)
Supplement: Supporting Information S1 — Experimental replicates and statistics Figure 1A and B (0.03 MB DOC) [file pone.0004953.s001.doc]

Supplementary Table 1: Figure 1A

| Experiment Replicate | Serum control  Mean ± SD | uRBC  Mean ± SD | CS2 PE  Mean ± SD | Other PE  Mean ± SD | P value |
| --- | --- | --- | --- | --- | --- |
| 1 | 15.4 ± 2.1 | 15.6 ± 0.4 | 43.6 ± 4.8 |  | 0.0006 |
| 2 | 81.1 ± 7.2 | 92.8 ± 4.0 | 110.0 ± 1.5 |  | 0.0163 |
| 3 | 71.5 ± 4.7 | 76.0 ± 0.7 | 88.5 ± 3.5 |  | 0.0037 |
| 4 | 7.2 ± 2.0 | 7.1 ± 0.7 | 24.8 ± 2.5 | 26.2 ± 4.1 | 0.0001- CS2  0.0005- E8B |
| 5 |  | 10.4 ± 13.4 |  | 31.2 ± 16.6 | 0.0189- ITG |

Concentration in ng/mL

* Statistics: Student’s t-test performed on uRBC vs PE

Supplementary Table 2: Figure 1B

| Experiment Replicate | Isotype control  Mean ± SD | Isotype GPI  Mean ± SD | CD88 Control  Mean ± SD | CD88 GPI  Mean ± SD | P value |
| --- | --- | --- | --- | --- | --- |
| 1 | 0.25 ± 0.02 | 1.96 ± 0.08 | 0.35 ± 0.04 | 4.72 ± 0.72 | 0.0190 |
| 2 | 0.17 ± 0.13 | 0.69 ± 0.18 | 1.023 ± 0.35 | 2.63 ± 2.63 | 0.0279 |

* Statistics: Student’s t-test performed on CD88 Control – Isotype Control vs. CD88 GPI – Isotype GPI
