# Supplementary material for: C5a Enhances Dysregulated Inflammatory and Angiogenic Responses to Malaria In Vitro: Potential Implications for Placental Malaria
Source: PLoS One. 2009 Mar 24;4(3):e4953. doi: 10.1371/journal.pone.0004953 (PMC2655724; doi:10.1371/journal.pone.0004953)
Supplement: Supporting Information S2 — Experimental replicates and statistics Figure 2B (TNF) (0.03 MB DOC) [file pone.0004953.s002.doc]

Supplementary Table 3: Figure 2

| Experiment Replicate | Concentration C5a (nM) | TNF Media  Mean ± SD | TNF GPI Mean ± SD | P value (C5a*GPI) |
| --- | --- | --- | --- | --- |
| 1 | 0  1  5  10  50  100 | 1. ± 0.0   0.0 ± 0.0  0.0 ± 0.0  0.0 ± 0.0  0.0 ± 0.0  0.0 ± 0.0 | 50.7 ± 1.7  76.4 ± 1.8  90.8 ± 33.1  180.6 ± 19.7  228.5 ± 6.3  348.6 ± 44.6 | <0.0001 |
| 2 | 0  1  5  10  50  100 | 0.0 ± 0.0  0.0 ± 0.0  0.0 ± 0.0  0.0 ± 0.0  0.0 ± 0.0  0.0 ± 0.0 | 9.0 ± 9.9  13.2 ± 15.8  24.5 ± 11.3  25.3 ± 13.5  52.9 ± 9.4  110.7 ± 57.1 | 0.0169 |
| 3 | 0  1  5  10  50  100 | 108.2 ± 78.2  69.2 ± 18.2  49.5 ± 14.5  38.1 ± 9.4  32.1 ± 10.1  70.6 ± 52.3 | 463.3 ± 36.0  460.1 ± 102.7  483.8 ± 38.3  600.2 ± 171.2  661.0 ± 162.5  934.1 ± 114.2 | 0.0003 |

Concentration in pg/mL

Synergism- 2-way ANOVA (C5a*GPI)
