# Supplementary material for: C5a Enhances Dysregulated Inflammatory and Angiogenic Responses to Malaria In Vitro: Potential Implications for Placental Malaria
Source: PLoS One. 2009 Mar 24;4(3):e4953. doi: 10.1371/journal.pone.0004953 (PMC2655724; doi:10.1371/journal.pone.0004953)
Supplement: Supporting Information S3 — Experimental replicates and statistics Figure 3 (0.04 MB DOC) [file pone.0004953.s003.doc]

Supplementary Table 4a: Figure 3A (IL-6)

| Experiment Replicate | Media  Mean ± SD | C5a  Mean ± SD | GPI  Mean ± SD | C5a + GPI Mean ± SD | P value (C5a*GPI) |
| --- | --- | --- | --- | --- | --- |
| 1 | 110.8 ± 30.9 | 4604± 4048 | 3749 ± 1055 | 20699 ± 252 | 0.0137 |
| 2 | 9.06 ± 15.7 | 0.0 ± 0.0 | 4528 ± 984 | 28701±3722 | <0.0001 |
| 3 | ** values over CBA std curve, synergism confirmed by elisa at 24 hours P=0.0003 | | | | |

Concentration in pg/mL

Supplementary Table 4b: Figure 3B (TNF)

| Experiment Replicate | Media  Mean ± SD | C5a  Mean ± SD | GPI  Mean ± SD | C5a + GPI  Mean ± SD | P value (C5a*GPI) |
| --- | --- | --- | --- | --- | --- |
| 1 | 0.0 ± 0.0 | 63.0± 89.2 | 164.1± 53.0 | 3793 ± 123 | <0.0001 |
| 2 | 0.0 ± 0.0 | 0.0 ± 0.0 | 3073 ± 999 | 6253 ± 1393 | 0.0124 |
| 3 | 4.8 ± 3.7 | 227.0 ± 64.0 | 97.0 ± 21.0 | 1630 ± 491 | 0.0002 |

Concentration in pg/mL

Supplementary Table 4c: Figure 3C (IL-1ß)

| Experiment Replicate | Media  Mean ± SD | C5a  Mean ± SD | GPI  Mean ± SD | C5a + GPI  Mean ± SD | P value (C5a*GPI) |
| --- | --- | --- | --- | --- | --- |
| 1 | 10.9 ± 15.5 | 1043 ± 691 | 1499 ± 432 | 1043 ± 691 | <0.0001 |
| 2 | 0.0 ± 0.0 | 29.1 ± 25.7 | 12.3 ± 21.3 | 276.6 ± 164.8 | 0.0418 |
| 3 | 2408 ± 614 | 2273 ± 346 | 2229 ± 110 | 7012 ± 3024 | 0.0081 |

Concentration in pg/mL

Supplementary Table 4d: Figure 3D (IL-10)

| Experiment Replicate | Media  Cumulative cytokines (Mean ± SD) | C5a  Cumulative cytokines (Mean ± SD) | GPI  Cumulative cytokines (Mean ± SD) | C5a + GPI  Cumulative cytokines (Mean ± SD) | P value (C5a*GPI) |
| --- | --- | --- | --- | --- | --- |
| 1 | 0.0 ± 0.0 | 22.7 ± 32.1 | 250.0 ± 22.0 | 724.6 ± 69.0 | 0.0013 |
| 2 | 0.0 ± 0.0 | 0.0 ± 0.0 | 56.1 ± 33.5 | 552.0 ± 56.0 | <0.0001 |
| 3 | 1331 ± 57.8 | 1740 ± 49.0 | 1131 ± 143 | 2009 ± 124.5 | 0.0006 |

Concentration in pg/mL

* Used cumulative cytokine production over time course, two-way ANOVA, interaction effect

Supplementary Table 4e: Figure 3E-H

| Experiment Replicate | Isotype  Cumulative cytokines (Mean ± SD) | Anti-CD88  Cumulative cytokines (Mean ± SD) | P value (Isotype vs. Anti-CD88) |
| --- | --- | --- | --- |
| 1 | IL-6: 24678 ± 3099  TNF: 5893 ± 421  IL-1ß: 269. 3 ± 70.2  IL-10: 374.8 ± 30.3 | IL-6: 1603 ± 2634  TNF: 929 ± 1693  IL-1ß: 61.5 ± 106.6  IL-10: 274.8 ± 34.0 | 0.0005  0.0028  0.0479  0.0191 |
| 2 | IL-6: 24678 ± 3099  TNF: 5893 ± 421  IL-1ß: 269. 3 ± 70.2  IL-10: 374.8 ± 30.3 | IL-6: 7154 ± 671.3  TNF: 18.7 ± 1.2  IL-1ß: 2968 ± 195  IL-10: 186.9 ± 41.5 | 0.0130  <0.0001  0.0039  0.0023 |

Concentration in pg/mL

* Repeated measures ANOVA comparing Isotype vs. anti-CD88 C5a and GPI co-treated cells corrected for background levels observed in media controls.
