# Supplementary material for: C5a Enhances Dysregulated Inflammatory and Angiogenic Responses to Malaria In Vitro: Potential Implications for Placental Malaria
Source: PLoS One. 2009 Mar 24;4(3):e4953. doi: 10.1371/journal.pone.0004953 (PMC2655724; doi:10.1371/journal.pone.0004953)
Supplement: Supporting Information S4 — Experimental replicates and statistics Figure 4 (0.05 MB DOC) [file pone.0004953.s004.doc]

Supplementary Table 5: Figure 4A-E Synergism

| Experiment Replicate | Media  Mean ± SD | C5a  Mean ± SD | GPI  Mean ± SD | C5a + GPI  Mean ± SD | P value (C5a*GPI) |
| --- | --- | --- | --- | --- | --- |
| 1 | MIP1α:  37.7 ± 20.3  MIP1β:   1. ± 0.0   IL-8   1. ± 0.0   MCP-1  5.3± 10.7 | MIP1α:   1. ± 0.0   MIP1β:   1. ± 0.0   IL-8  99.4 ± 198.9  MCP-1  942 ± 772 | MIP1α:  586.2 ± 190.8  MIP1β:  2748 ± 759.8  IL-8  85254 ± 69846  MCP-1  4537 ± 1900 | MIP1α:  6695 ± 3902  MIP1β:  11633 ± 5641  IL-8  320000 ± 0.0¶  MCP-1  4615 ± 2270 | 0.0084  0.0088  <0.0001  0.5847 |
| 2 ** | MIP1α:  0.0 ± 0.0  MIP1β:  0.0 ± 0.0  IL-8:  18867 ± 1466  MCP-1  10466 ± 1258 | MIP1α:  0.0 ± 0.0  MIP1β:  0.0 ± 0.0  IL-8:  38083 ± 762  MCP-1  55889 ± 6823 | MIP1α:  0.0 ± 0.0  MIP1β:  0.0 ± 0.0  IL-8:  50284 ± 2871  MCP-1  34436 ± 8775 | MIP1α:  421.6 ± 98.44 MIP1β:  2288 ± 705.0  IL-8:  299149 ± 57459  MCP-1  108810 ± 1778 | 0.0038  0.0101  0.0022  0.0225 |
| 3 | MIP1α:  21.5 ± 19.9  MIP1β:  120.6 ± 86.7  IL-8:  1.9 ± 2.1  MCP:  10.2 ± 0.3  sFlt-1:  0.003 ± 0.001 | MIP1α:  10.0 ± 0.0  MIP1β:  24.0 ± 24.3  IL-8:  34.0 ± 2.9  MCP-1  17.3 ± 3.75  sFlt-1:  0.002 ± 0.002 | MIP1α:  80.9 ± 8.8  MIP1β:  186.2 ± 37.6  IL-8:  95.9 ± 25.2  MCP-1  33.7 ± 12.5  sFlt-1:  0.006 ± 0.002 | MIP1α:  747.6 ± 108.1  MIP1β:  3723 ± 188.4  IL-8:  286.9 ± 98.1  MCP-1  176.4 ± 168.9  sFlt-1:  0.022 ± 0.002 | <0.0001  <0.0001  0.0265  0.0092*  <0.0001*** |
| 4 | sFlt-1:  210.4 ± 41.8 | sFlt-1:  190.6 ± 150 | sFlt-1:  535.2 ± 72.0 | sFlt-1:  1966 ± 280 | 0.0034 |
| 5 | sFlt-1:  48.2 ± 15.8 | sFlt-1:  187.0 ± 78.6 | sFlt-1:  359.3 ± 127.9 | sFlt-1:  949.9 ± 145.6 | 0.0010 |
| 6 | sFlt-1:  41.3 ± 21.2 | sFlt-1:  66.0 ± 29.1 | sFlt-1:  148.9 ± 33.5 | sFlt-1:  308.8 ± 0.0 | 0.0177 |

Concentration in pg/mL unless otherwise indicated

Synergism: Two-way ANOVA interaction term, * log transformed data to normalize variance

¶ Saturated Elisa plate

** Experiment on purified monocytes

*** ODs

Supplementary Table 6: Figure 4A-E Receptor blockade

| Experiment Replicate | Isotype (C5a + GPI)  Mean ± SD | Anti-CD88 (C5a + GPI)  Mean ± SD | P value (Isotype vs. Anti-CD88) |
| --- | --- | --- | --- |
| 1 | MIP1α: 2773 ± 237  MIP1β: 8484 ± 1279  IL-8: 2.0 ± 0.2  MCP-1: 8580 ± 1082  sFlt-1: 74.5 ± 5.9 | MIP1α: 0.0 ± 0.0  MIP1β: 526.6 ± 322  IL-8: 0.5 ± 0.3*  MCP-1: 6795 ± 899  sFlt-1: 0.0 ± 0.0 | 0.0002 ¥  <0.0001  0.0002*  0.0443  0.0359 ¥ |
| 2 | MIP1α: 280.4 ± 1.1  MIP1β: 4003.6 ± 149.7  IL-8: 27567 ± 11146  MCP-1: 34938 ± 4960  sFlt-1: 226.5 ± 92.1 | MIP1α: 0.0 ± 0.0  MIP1β: 345.5 ± 76.1  IL-8: 3337 ± 7558  MCP-1:19937 ± 4132  sFlt-1: 121.1 ± 21.6 | <0.0001 ¥  0.0011  0.1259  0.0815  0.3853 |
| 3 | MIP1α: 672.8 ± 102.2  MIP1β: 3160 ± 391.3  IL-8: 231.4 ± 228.7  MCP-1: 110.7 ± 44.1  sFlt-1: 0.015 ± 0.008 | MIP1α: 39.4 ± 33.2  MIP1β: 179.4 ± 113.9  IL-8: 108.1 ± 71.61  MCP-1: 31.8 ± 9.1  sFlt-1: 0 ± 0.0 | 0.0005  0.0002  0.4231  0.0388  0.0869 ¥ |

Concentration in pg/mL unless otherwise indicated

Blockade- Student’s t-test comparing Isotype vs. anti-CD88 C5a and GPI co-treated cells corrected for background levels observed in media controls

¥ One sample t-test comparing Isotype to hypothetical “0”

* ODs
